# iKcr-DRC: prediction of lysine crotonylation sites in proteins based on a novel attention module and DenseNet

**DOI:** 10.3389/fgene.2025.1574832

**Published:** 2025-06-11

**Authors:** Xin Wei, Siqin Hu, Jian Tu, Muhammad Akmal Remli

**Affiliations:** ^1^ Institute for Artificial Intelligence and Big Data, Universiti Malaysia Kelantan, Kota Bharu, Kelantan, Malaysia; ^2^ Business School, Jiangxi Institute of Fashion Technology, Nanchang, China; ^3^ Faculty of Data Science and Computing, Universiti Malaysia Kelantan, Kota Bharu, Kelantan, Malaysia; ^4^ School of Mega Data, Jiangxi Institute of Fashion Technology, Nanchang, China

**Keywords:** protein post-translational modification, lysine crotonylation site, deep learning, DenseNet, channel attention mechanism

## Abstract

**Introduction:**

Lysine crotonylation (Kcr) is a recently identified post-translational modification that predominantly occurs on lysine residues and plays a crucial role in regulating gene expression, cellular metabolism, and various biological processes. Increasing evidence has linked Kcr to the pathogenesis of major diseases such as cancer, highlighting the importance of accurately identifying Kcr sites for understanding disease mechanisms and normal cellular function.

**Methods:**

In this study, we present a novel deep learning-based computational model, named iKcr-DRC, for the accurate prediction of lysine crotonylation sites. The model leverages a densely connected convolutional network (DenseNet) as its backbone to effectively capture high-level local features from protein sequences. Additionally, we introduce an enhanced channel attention mechanism with a short-circuit connection design, endowing the network with residual properties and improved feature refinement capabilities.

**Results:**

The experimental results show that the iKcr-DRC model achieves 90.30%, 78.35%, 84.33% and 69.15% for sensitivity, specificity, accuracy, and Matthew’s correlation coefficients, respectively. These results indicate a significant improvement over existing state-of-the-art Kcr prediction tools.

**Discussion:**

The proposed iKcr-DRC model provides an effective and innovative approach for predicting lysine crotonylation sites. It holds great potential for advancing applications in bioinformatics and enhancing the understanding of protein post-translational modifications. An online prediction tool based on the iKcr-DRC model is freely accessible at: http://www.lzzzlab.top/ikcr/.

## 1 Introduction

Protein post-translational modifications (PTMs) (Hu et al., 2023) are widespread physiological phenomena in organisms and play an important role in the functional regulation of cells (Kouzarides, 2007; Verdin and Ott, 2015). Currently, modern technology has discovered more than 600 different types of PTMs (Meng et al., 2022), which include a variety of modifications such as phosphorylation, glycolysis, acetylation and sacculation (Macek et al., 2019). These modifications can change protein structure, function, and interaction, thus producing important regulatory effects on biological processes within the cell (Fu et al., 2018). Protein lysine crotonylation (Kcr) (Tan et al., 2011) is an important PTM modality that occurs on lysine residues. Catalyzed by acyltransferases, acetyl groups are added to residues of lysine, thereby changing protein chemical structure and function. Kcr plays an important role in several cells processes, including key processes such as gene transcription and cellular metabolism (Patel et al., 2024). It is involved in the regulation of these cells processes by modulating protein interactions, activity, and stability (Hou et al., 2021). Medical research has shown that Kcr plays an important regulatory role in major diseases such as tumors and cancer (Gao et al., 2023; Jiang et al., 2021). Therefore, accurate prediction of the Kcr site is critical for understanding an organism’s normal function.

In the past, In order to explore the importance of Kcr sites in biological processes, researchers have developed a number of biological experimental methods (Yu et al., 2020) to identify Kcr sites. However, these methods suffer from high costs. Therefore, researchers urgently need to develop more convenient and efficient computational methods to replace the traditional biomedical experimental methods. Computational methods enable the prediction of Kcr sites, while biological experiments provide validation and contextual support for these predictions. In recent years, researchers have developed a series of compelling Kcr site models. These models can accurately predict Kcr sites of proteins, thus helping researchers to further explore the functions and regulatory mechanisms of protein modifications in biology. In 2016, researchers created a small dataset of Kcr sites and applied a discrete hidden Markov model (DHMM) to predict them (Qiu et al. 2017). Subsequently, many researchers (Ju and He, 2017; Liu et al., 2020; Malebary et al., 2019; Qiu et al., 2017; Qiu et al., 2018; Wang et al., 2020) have worked based on this dataset. In 2017, Qiu et al. (2017) developed a model called Position-weight. The model encodes the Kcr site sequence using position-weighted amino acid composition (PWAA) (Ismail et al., 2016) and predicts it by support vector machine (SVM). The results of the study show that the model exhibits good performance in prediction. In the same year, Ju and He (2017) developed a model called CKSAAP-CrotSite, which utilizes k-spaced amino acid pairs (CKSAAP) and SVM to encode and predict Kcr sites. In 2019, the iKcr-PseEns model (Qiu et al., 2018) and the iCrotoK-PseAAC model (Malebary et al., 2019) used Random Forest and Artificial Neural Network (ANN) to predict Kcr sites, respectively. These two models employ different algorithms and feature coding methods to further improve the accuracy and performance of the Kcr site prediction models. In 2020, Liu et al. (2020) developed a model called LightGBM-CroSite. The model encodes Kcr site sequences with multiple complicated coding methods and uses the LightGBM algorithm for prediction. Experimental results showed that the model demonstrated the best prediction results. These models performed well in predicting the Kcr site, especially the iCrotoK-PseAAC and LightGBM-CroSite models, which had a prediction accuracy of 99%. However, machine learning predictive models usually perform well on small datasets, but they are relatively less innovative. In addition, these machine learning models are highly dependent on complex feature encoding methods to improve predictive performance, yet the complexity of feature coding is a rather difficult task. All these limitations are drawbacks of current machine learning models.

Deep learning models (Sun et al., 2022; Wang T. et al., 2023; Wang et al., 2022; Zhang L. et al., 2021) show great potential in a big data-driven context. In 2020, Lv et al. (2021a) created a balanced benchmark dataset of Kcr sites, while they developed a predictive model called Deep-Kcr by using a convolutional neural network. In 2021, Qiao et al. (2022) based on this dataset, they encoded the Kcr site sequences by the BERT model (Le et al., 2021) and utilized the BiLSTM network (Sharma et al., 2022) to capture the global features of the Kcr site sequences, so as to extract richer feature information for predicting the Kcr site. Experimental results show that this model achieves good results. In the same year, Khanal et al. (2022) introduced capsule network (Chen Z. et al., 2023) to replace the traditional convolutional neural network, because the traditional convolutional neural network may lose the spatial information when compressing the feature information, and the introduction of capsule network further improved the experimental results. However, the main network structures adopted by these prediction models are too basic and lack innovation, and thus there is still a wide scope of exploration in the field of deep learning methods.

We propose a deep learning model called iKcr-DRC, which employs DenseNet (Wei et al., 2022) as the core network structure to extract advanced local feature information. Traditional convolutional neural networks pass feature information from layer by layer, whereas DenseNet networks use a densely connected structure that allows each layer to be directly connected to all previous layers, thus effectively utilizing the feature map information of the previous layers. This dense connection structure allows our model to avoid network degradation problems and extract richer feature information. In addition, we have made innovative improvements to the channel attention mechanism (Chen W. et al., 2023; Meng et al., 2023) by introducing the design of short-circuit connections, which makes the channel attention mechanism internally equipped with residual structures. The improved channel attention mechanism can more accurately compute the channel weights of the output feature maps of the DenseNet network, thus improving the performance and accuracy of the model. With these improvements, our iKcr-DRC model shows better performance in the Kcr site prediction task. This study provides new ideas and methods for the application of deep learning in bioinformatics.

## 2 Materials and methods

### 2.1 Benchmark dataset

In this study, we used the benchmark dataset created by Lv et al. (2021b). The protein sequences in this dataset were extracted from the UniProt database (UniProt Consortium, 2011), and to avoid redundancy, they applied the CD-HIT tool (Huang et al., 2010) to remove duplicate protein sequences with more than 30% similarity. In the end, they obtained 9,964 samples of real Kcr site and 9,964 samples of spurious Kcr site. Based on experimental confirmation, the appropriate length for positive and negative samples is 29, and they are both centered at the K site. In positive samples, K indicates crotonylation, while in negative samples, K indicates no crotonylation modification. Finally, they randomly split the positive and negative samples into a training set and an independent test set in a ratio of 7:3. The final training dataset contained 6,975 Kcr site samples and 6,975 non-Kcr site samples, while the independent test dataset contained 2,989 Kcr site samples and 2,989 non-Kcr site samples. To ensure the uniqueness of each sequence, different classes of protein sequences are mutually independent. The details of the benchmark dataset are shown in Table 1.

**TABLE 1:** The details of the benchmark dataset.

Original dataset	Positive	Negative
Training Dataset	6,975	6,975
Testing Dataset	2,989	2,989
Total	9,964	9,964

### 2.2 Feature extraction methods

There are some shortcomings in the current methods of protein feature coding. First, some feature coding methods encode protein sequences as fixed-length vectors or matrices, but these methods can be improved in capturing contextual information between proteins. Second, traditional coding methods rely on manual design, which leads to complex and difficult feature construction. In summary, these traditional methods have some limitations in protein feature coding.

Word embedding model (Ren et al., 2022) is a feature coding model based on contextual semantic information and shallow neural network training. In the field of bioinformatics, word embedding models are widely applied. Word embedding coding of protein sequences involves the following steps: first, the protein sequence is divided into K-mers of fixed length using a sliding window. Next, these k-mers are transformed to binary vectors using One-hot coding (Abbas et al., 2022). All binary vectors are trained using a two-layer neural network to generate the weight parameter matrix. Finally, this weight parameter matrix is multiplied with a binary vector to obtain a dense vector for representing K-mer words. By training the word embedding model, we can obtain a dense vector representation of each K-mer word, and it possesses contextual semantic relations. We set the sliding window size to 1, the fixed length to 1-mers, and use an 80-dimensional word vector to represent each 1-mer. Therefore, the length of each Krc site sequence is 29, which can be expressed as a feature matrix of size 80 × 29.

### 2.3 Model construction

**FIGURE 1:**
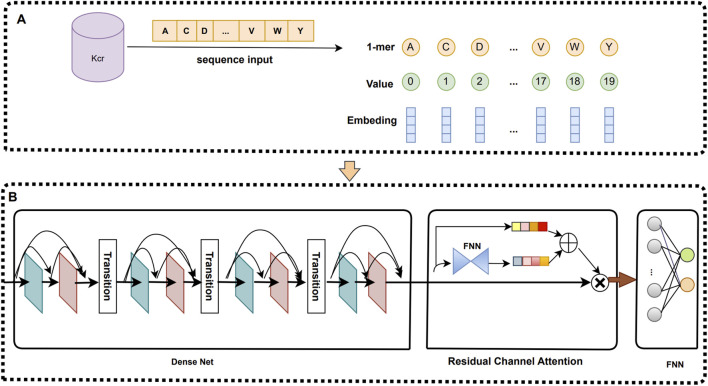
An overview of iKcr-DRC model. **(A)** Feature coding. **(B)** iKcr-DRC network framework.

In this study, we propose a deep learning prediction model called iKcr-DRC, which is capable of automatically coding protein sequences and extracting high-level feature information to improve the prediction accuracy. First, we apply a word embedding technique to each Krc site sequence and convert it into a feature encoding matrix of size 80 × 29. Then, we directly input this feature encoding matrix into the DenseNet network with Dense Connectivity to fully explore the deep features of the sequences. Subsequently, we introduce the residual channel attention mechanism on the high-level feature maps extracted by DenseNet, which further enhances the representation of the feature maps by emphasizing the importance of the information of each channel. Finally, we input the feature maps evaluated by the attention mechanism into the fully connected neural network for prediction. Figure 1 illustrates the specific details of this network framework.

#### 2.3.1 DenseNet

In this study, we make structural improvements based on the standard DenseNet framework. The traditional DenseNet usually contains an initial convolutional layer, multiple Dense blocks, and several Transition layers, where low-level features are first extracted from the initial convolutional layer, and then input into the Dense blocks and Transition layers to obtain richer high-level representations. In our improved scheme, the initial convolutional layer is first eliminated, and the feature coding matrix is directly input into the Dense blocks to fully utilize the information of the original data. At the same time, we also add an additional batch normalization layer (Berrar and Dubitzky, 2021) between each Dense block and Transformation layer, which improves the robustness and stability of the model while reducing its complexity.

##### 2.3.1.1 Dense block

Dense block (Yang et al., 2020) is a key component of DenseNet network and it plays an important role. The Dense block consists of multiple densely connected convolutional layers, where the output of each convolutional layer is connected to the outputs of all previous convolutional layers to produce a densely connected network structure. The Dense block equation is shown below.

$$x_{l}=F_{l}\left(\left[x_{0},x_{1},\cdots ,x_{l-1}\right]\right),l=1,2,\cdots ,L.$$ (1)

$$F_{l}\left(x\right)=Conv\left(ReLU\left(BN\left(x\right)\right)\right)$$ (2)

where $x_{l}$ denotes the concatenation operation in the feature channel dimension and F is a composite function consisting of the batch normalization (BN), ReLU activation function and convolution (Conv) operation.

**FIGURE 2:**
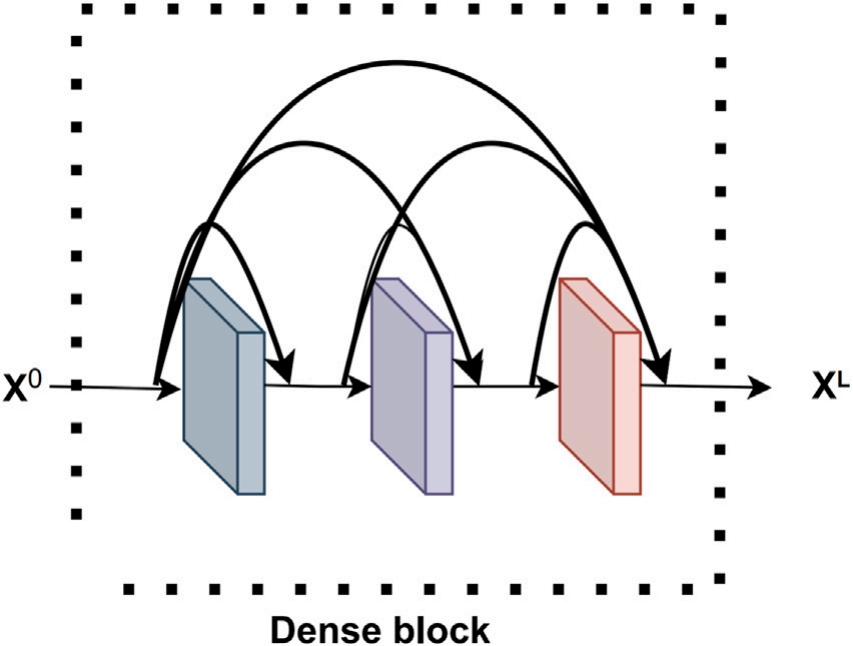
The structure of Dense block.

This densely connected structure can effectively integrate shallow features with deeper features, thus realizing the reuse of feature information. The Dense block structure improves the overall performance of the model in terms of gradient flow, computational efficiency, and model generalization ability, enabling iKcr-DRC to perform Krc site prediction more accurately. The Dense block structure is shown in Figure 2.

##### 2.3.1.2 Dense block

The Transition layer (Jia et al., 2023a) in the DenseNet network is mainly designed to reduce the size of the feature map and thus control the complexity of the model. The Transition layer is located between adjacent Dense blocks and consists of a convolutional layer (1 × 1) and an average pooling layer (2 × 2). The parameters of the Dense block’s output feature maps are huge, which can lead to parameter explosion and slower training. Therefore, the Transition layer uses a convolutional layer (1 × 1) to reduce channel number of the feature map and an average pooling layer (2 × 2) to compress the spatial size of the feature map. This reduces the parameters of the feature map and reduces the complexity of the model, thus improving the generalizability of the model.

We add a batch normalization layer to the Transition layer. The batch normalization layer can normalize the input data to optimize and accelerate the model training and improve the model performance. The Transition layer equation is shown below.

$$x_{trans}=T\left(x_{block}\right)=AvgPool\left({Conv}_{1\times 1}\left(BN\left(\left[x_{0},\cdots ,x_{L}\right]\right)\right)\right)$$ (3)

#### 2.3.2 Residual channel attention mechanisms

**FIGURE 3:**
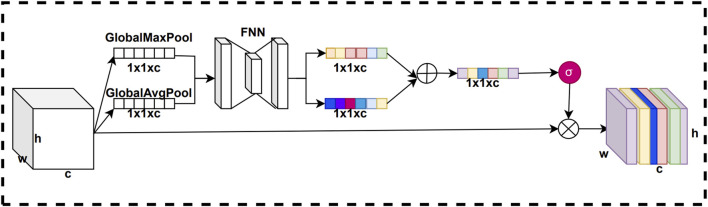
Structure of the channel attention mechanism.

In convolutional neural networks, the weights of each channel of the feature map are fixed, and there is no adaptive learning based on the importance of the features. However, the importance of feature information is not the same for different channels. By using the channel attention mechanism, we can adaptively weight the channel feature information according to its importance, thus enhancing the attention of the model on important feature information. This improves the representation of features, which in turn improves the performance and generalization of the model. The structure of the channel attention mechanism is shown in Figure 3.

We assume that in the channel attention mechanism, useless feature information is already filtered when the feature map is dimensionally compressed using the Pooling layer. In a way, the feature vectors obtained by pooling layer compression already has the potential role of channel attention weights. Based on this assumption, we improve the channel attention mechanism by introducing a short-circuit connection structure to improve its performance. The improved channel attention mechanism is called residual channel attention mechanism. The residual channel attention mechanism equation is shown below.

$$M_{c}\left(F\right)=\sigma \left(FNN\left(AvgPool\left(F\right)\right)+FNN\left(MaxPool\left(F\right)\right)\right)$$ (4)

$$M_{r}\left(F\right)=\sigma \left(AvgPool\left(F\right)+MaxPool\left(F\right)\right)$$ (5)

$$M_{rc}\left(F\right)=M_{c}\left(F\right)+M_{r}\left(F\right)$$ (6)

$$F=F_{scale}\left(F,M_{rc}\left(F\right)\right)=M_{rc}\left(F\right)\cdot F$$ (7)

where the pooling here is global max pooling and global average pooling. $M_{rc}\left(F\right)$ denotes the residual weight value. $F_{scale}$ denotes each channel specific value F multiplied by the weight.

**FIGURE 4:**
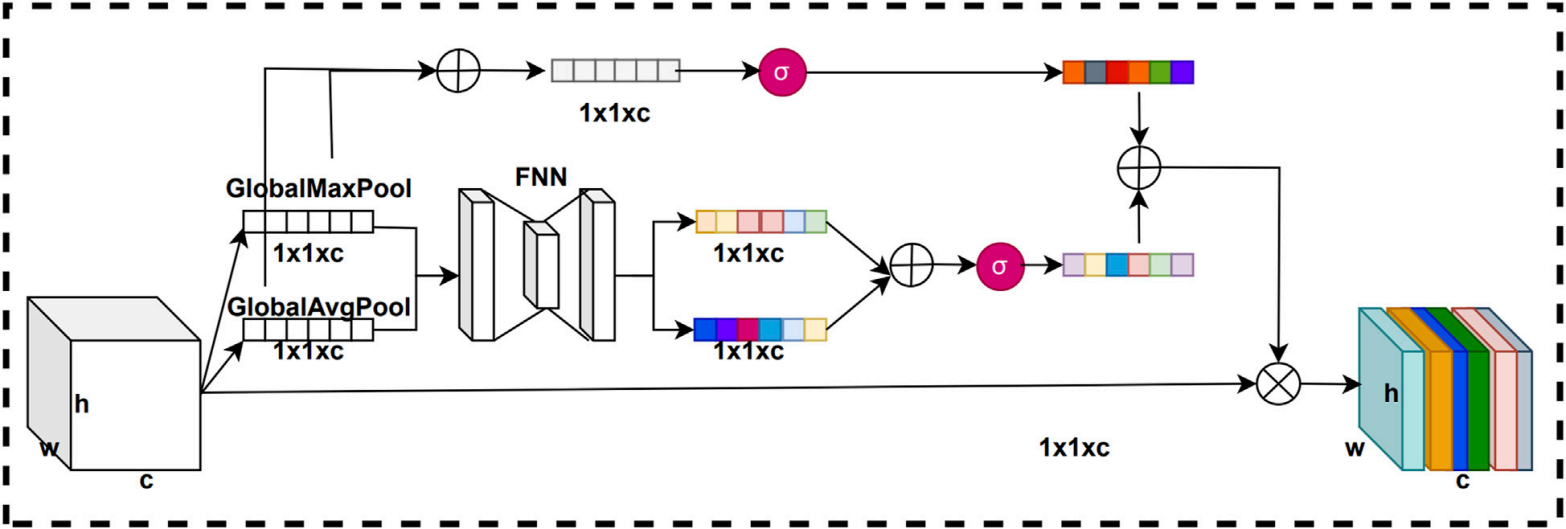
Structure of the residual channel attention mechanism.

In this residual channel attention mechanism, we add the feature vectors compressed by the pooling layer with the weight vectors obtained from the network training. In this way, the generated channel weight vectors can more accurately represent the importance of each channel in the feature map, thus improving representation of key feature information by the model. The purpose of this improvement is to make the network more attentive to important channel features and thus improve the performance of the model. The structure of the residual channel attention mechanism is shown in Figure 4.

The original channel attention mechanism first uses global max pooling and global average pooling compresses on the feature map to obtain pooled vectors of two channel dimension sizes. Next, we input these two pooled vectors into a fully connected neural network for training to get two trained vectors. Then, we sum these two training vectors to get a summation vector. Finally, the summation vector is normalized using the sigmoid activation function to obtain the channel attention weights. To improve the channel attention mechanism, we introduce a short-circuit connection structure. First, we add the two pooling vectors to obtain a summation pooling vector. Next, we process the summation pooling vector using a sigmoid activation function to obtain a residual weight vector. Finally, the residual weight vector is summed with the channel attention weights to obtain the final residual channel attention weights.

#### 2.3.3 Fully connected neural network

We use DenseNet and residual channel attention mechanism as advanced feature extraction methods. With these methods, we can obtain a set of advanced feature representations. Next, the extracted advanced features are flattened into vectors and fed into a Fully connected neural network. Finally, we employ the softmax function (Duhan et al., 2022) to predict classification results.

### 2.4 Performance evaluation

To evaluate the predictive performance of the model more comprehensively, we used four common scientific evaluation metrics for the assessment. These evaluation metrics include sensitivity (Sn), specificity (Sp), accuracy (Acc) and Matthew’s correlation coefficient (MCC) (Jia et al., 2023b). By using these evaluation metrics, we can understand the experimental effect of the model in detail, which helps us to further improve and optimize the model. The evaluation metrics are as in Equation 8.

$$\left\{\begin{array}{l} Sp=\frac{TN}{TN+FP}\\ Sn=\frac{TP}{TP+FN}\\ Acc=\frac{TP+TN}{TP+TN+FP+FN}\\ MCC=\frac{TP\times TN-FP\times FN}{\sqrt{\left(TP+FP\right)\times \left(TP+FN\right)\times \left(TN+FP\right)\times \left(TN+FN\right)}} \end{array}\right.$$ (8)

Among them, True Positive (TP), True Negative (TN), False Positive FP) and False Negative (FN) are the four important indicators in the confusion matrix (Niu et al., 2021). These metrics represent the model’s accuracy in predicting positive and negative samples, respectively, and they are the basis for computing the evaluation metrics. To characterize the model performance more accurately, we also used ROC curves and AUC metrics (Jia et al., 2022) for evaluation. Higher values of all these evaluation metrics indicate better model performance.

## 3 Results and discussion

### 3.1 Comparing parameter combinations of dense blocks

**FIGURE 5:**
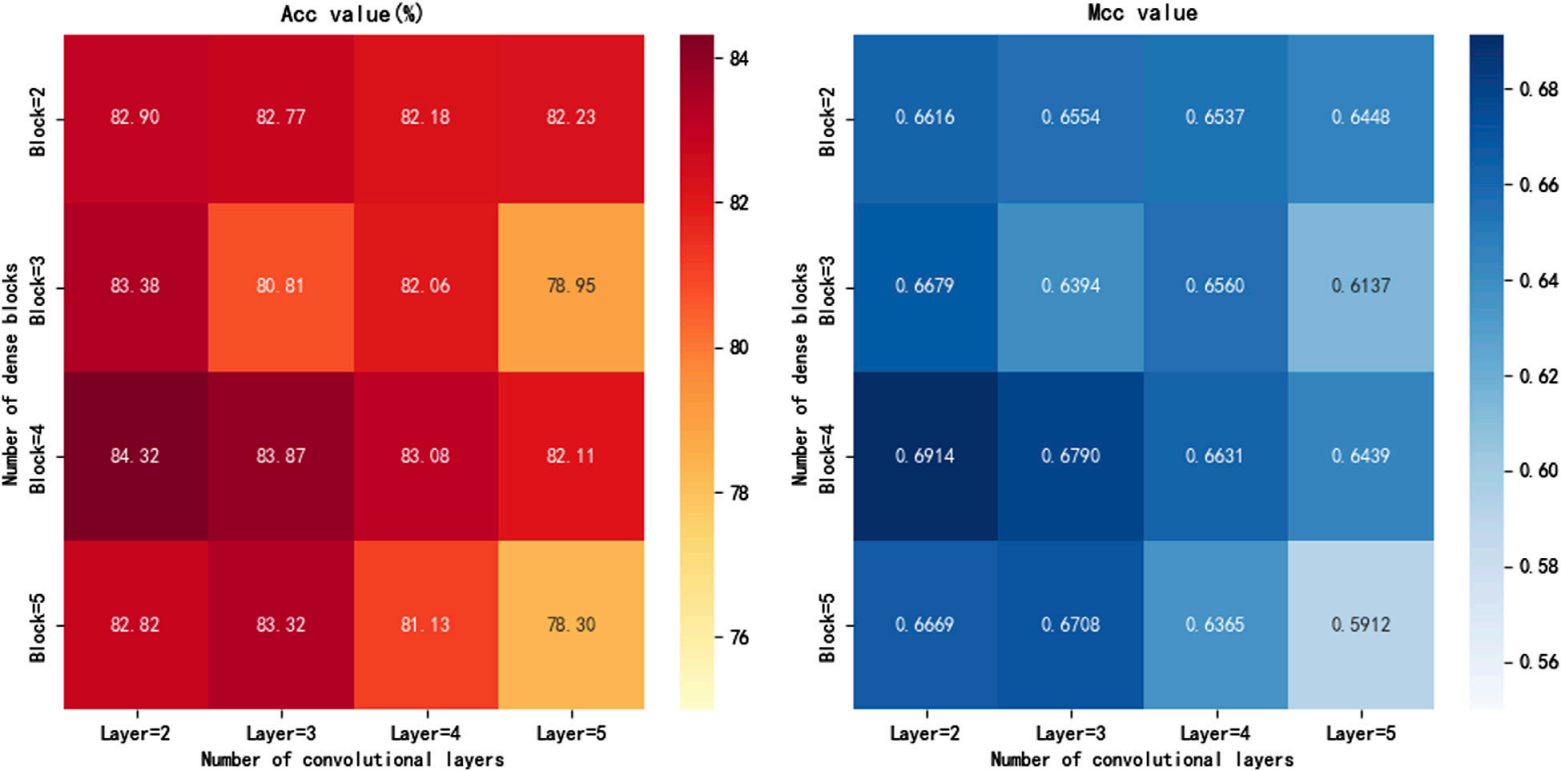
Evaluation metrics for different parameter of Dense blocks.

In this study, DenseNet serves as the core network structure of the iKcr-DRC model, and we conduct an in-depth exploration of the parameter combinations of the Dense block to effectively avoid overfitting while ensuring model performance. Specifically, we compare the model performance by adjusting the number of Dense blocks and the number of convolutional layers inside them. According to the experimental results in Figure 5, when the number of Dense blocks is set to four and the number of convolutional layers in each Dense block is set to 2, the Acc and MCC metrics of the model reach their maximum values. Based on this finding, we finally chose this parameter configuration to build the DenseNet network to achieve the best prediction results while balancing the complexity and performance.

### 3.2 Comparison of different coding methods

**FIGURE 6:**
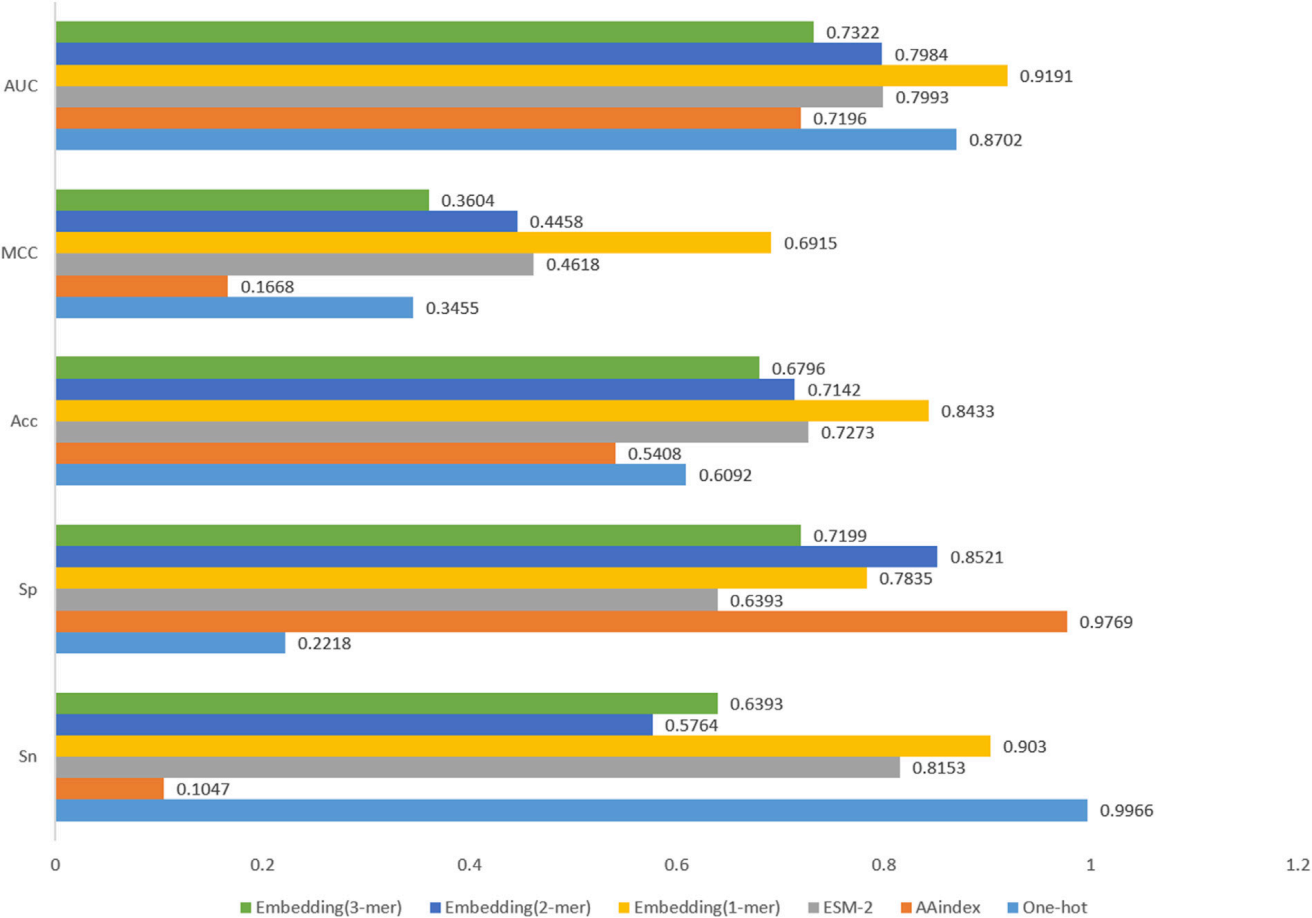
Comparative results of different coding schemes.

In this study, we compare several matrix feature coding methods because our model network structure can only accept feature matrices as input. These matrix feature coding methods include Word embedding, One-hot and AAindex (Wang X. et al., 2023). In addition, we discuss the application of Word embedding coding in three cases: 1-mer, 2-mer and 3-mer. We also compare the current popular protein large language model ESM-2. We input these matrix feature codes into the network framework and then compare the performance of the models. According to the experimental results in Figure 6, Word embedding coding has the highest value of all evaluation metrics when comparing the other three coding methods. The One-hot coding method has problems of sparsity and information loss, and the AAindex coding method mainly focuses on the amino acid composition of proteins while ignoring other key structural and functional information. The ESM-2 coding method generates embedding vectors of protein sequences based on a large-scale pre-trained language model, which is theoretically able to capture the deep semantic and structural information in the sequences. However, limited by computational resources, the ESM-2 model with the smallest parameters was chosen for encoding in this study, which led to its relatively poor performance in the experiments. In contrast, word embedding coding is a method of encoding word vectors based on contextual semantic information by network training, which is more efficient than manual feature coding. Meanwhile, we compare the experimental results of Word embedding coding in three cases (1-mer, 2-mer and 3-mer), and the results show that simple 1-mer obtains the best results. Therefore, we identified 1-mer Word embedding coding as the preferred scheme for feature coding.

### 3.3 Comparison of different attention mechanisms

In this study, we researched and analyzed the six attentional mechanisms. These six mechanisms include SENet (Li et al., 2023), channel attention mechanism (CAM), ECANet (Gong et al., 2023), CBAM (Jia et al., 2023c), DANet (Bai et al., 2023) and residual channel attention mechanism (RCAM). We combined these attention mechanisms with Dense Net and tested them. According to the results of the test experiments in Table 2, we can see that the RCAM shows better experimental results compared to the CAM. This suggests that by introducing a short-circuit connection structure into the CAM, the model can highlight more important channel feature information. Compared to simple SENet and CBAM, the RCAM is slightly better in terms of performance. The two latest attention mechanisms, ECANet and DANet, exhibit relatively poor performance, this is because complex attention mechanisms are usually only fully utilized on large-scale datasets. Synthesizing the experimental results, we finally chose the Residual Channel Attention Mechanism.

**TABLE 2:** Comparison of different attention mechanisms.

Model frameworks	Sn	Sp	Acc	MCC	AUC
SENet	0.8855	0.7785	0.8320	0.6679	0.9169
CAM	0.9203	0.7236	0.8220	0.6568	0.9126
ECANet	0.7460	**0.8909**	0.8185	0.6437	0.9106
CBAM	**0.9260**	0.7363	0.8312	0.6746	0.9181
DANet	0.7708	0.8728	0.8218	0.6470	0.9137
RCAM	0.9030	0.7835	**0.8433**	**0.6915**	**0.9191**

The highest score for each metric is highlighted by bold.

### 3.4 Comparison of different network frameworks

We researched the performance of multiple deep learning network frameworks in the task of Kcr site prediction. Specifically, we researched convolutional neural network models such as ResNet (Ge et al., 2022), DenseNet, InceptionResNet (Zhang X. et al., 2021), and EfficientNet (Dehghan Rouzi et al., 2023), and further experimented with two temporal network frameworks, BiGRU (Zhang et al., 2023) and Transformer (Zhou et al., 2022). In addition, we have tried the Vision Transformer (ViT) (Khedr et al., 2023) model, which is very compelling in the field of computer vision. According to the experimental results shown in Table 3, compared to using only the DenseNet network, the performance is significantly improved by adding the RCAM. InceptionResNet and EfficientNet are two newer deep neural network models that employ a series of complex structural designs to improve model performance. However, the simple DenseNet network structure shows better performance compared to these two complex convolutional neural networks. This indicates that the use of more complex network structures does not necessarily improve performance on small datasets. Similarly, we tried the BiGRU and Transformer models. These models showed good performance in the experiments, but they were slightly less effective relative to the combination of DenseNet and RCAM. ViT is a large model and requires more data to fully utilize its superior performance. However, in small data samples, the ViT network model cannot be utilized to its full potential. This indicates that by using DenseNet and the RCAM to extract features, we can obtain more advanced feature information, which makes it easier to predict the Kcr site. Based on the experimental results, we choose the combination of DenseNet and RCAM to build the final network framework.

**TABLE 3:** Comparison of different network architecture models.

Model frameworks	Sn	Sp	Acc	MCC	AUC
DenseNet	**0.9488**	0.6306	0.7897	0.6112	0.9021
ResNet + RCAM	0.8752	0.6814	0.7783	0.5674	0.8503
InceptionResNet + RCAM	0.9973	0.1063	0.5518	0.2283	0.8146
EfficientNet + RCAM	0.7142	0.6219	0.6681	0.3376	0.7148
BiGRU	0.8159	**0.8166**	0.8163	0.6326	0.8884
Transformer	0.8591	0.7969	0.8280	0.6573	0.9077
ViT	0.8390	0.7039	0.7714	0.5480	0.8438
DenseNet + RCAM	0.9030	0.7835	**0.8433**	**0.6915**	**0.9191**

The highest score for each metric is highlighted by bold.

### 3.5 Comparing different combinations of attention mechanisms and DenseNet networks

**FIGURE 7:**
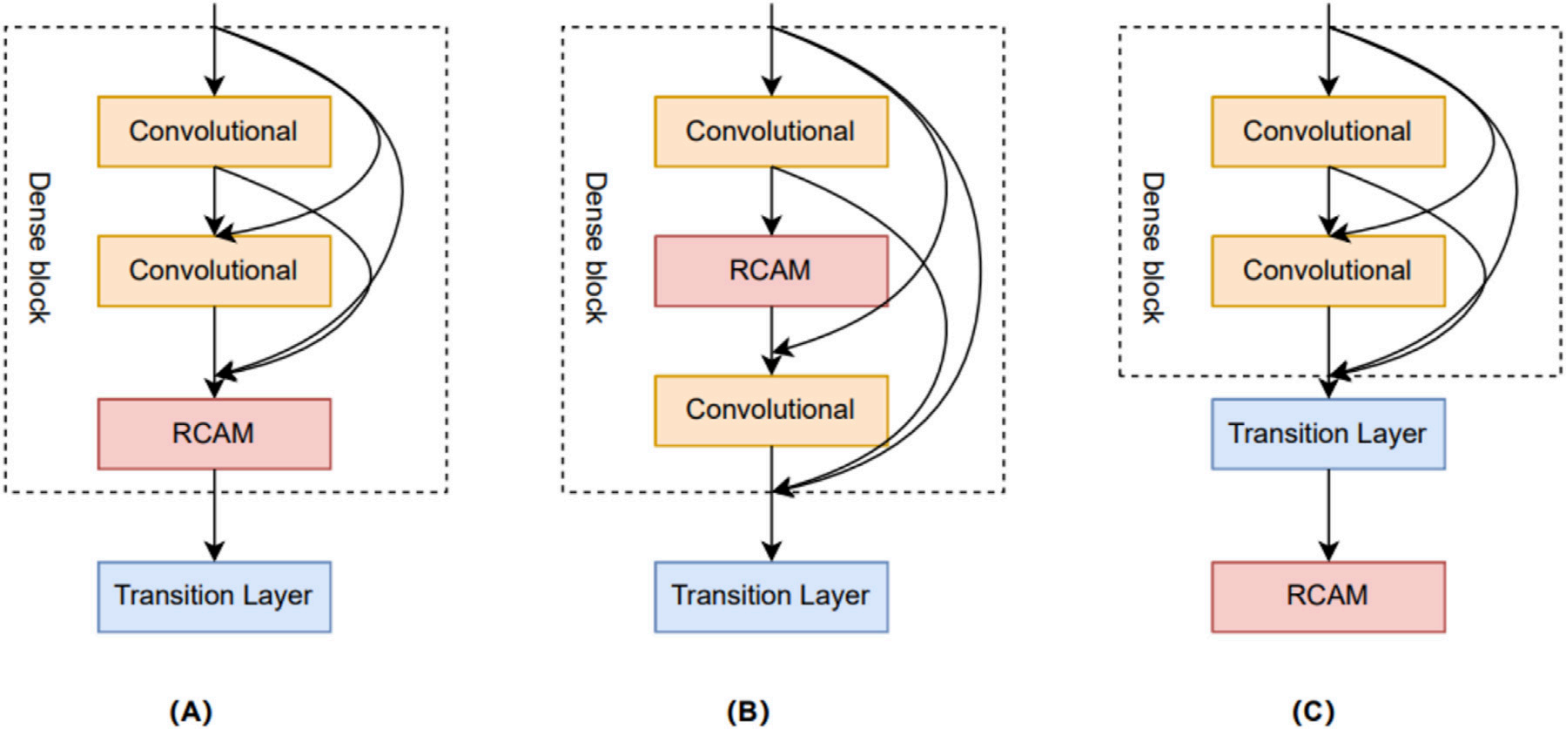
Three different network combination structures. **(A)** Dense + RCAM + Net. **(B)** Den + RCAM + seNet. **(C)** DenseNet + RCAM.

In this study, we delve into three different approaches for combining residual channel attention mechanisms with DenseNet networks. The goal of these combined approaches is to fully utilize the advantages of the residual channel attention mechanism and the DenseNet network to further enhance model performance and feature representation. These three different combinations are shown in Figure 7. The first combination approach is called Dense + RCAM + Net, which adds the residual channel attention mechanism between the Dense blocks and the Transient layer. The second combination approach is called Den + RCAM + seNet, which introduces the residual channel attention mechanism between the Convolutional layers of each Dense block. The third combination is called DenseNet + RCAM, which adds the residual channel attention mechanism after the final output of DenseNet.

**FIGURE 8:**
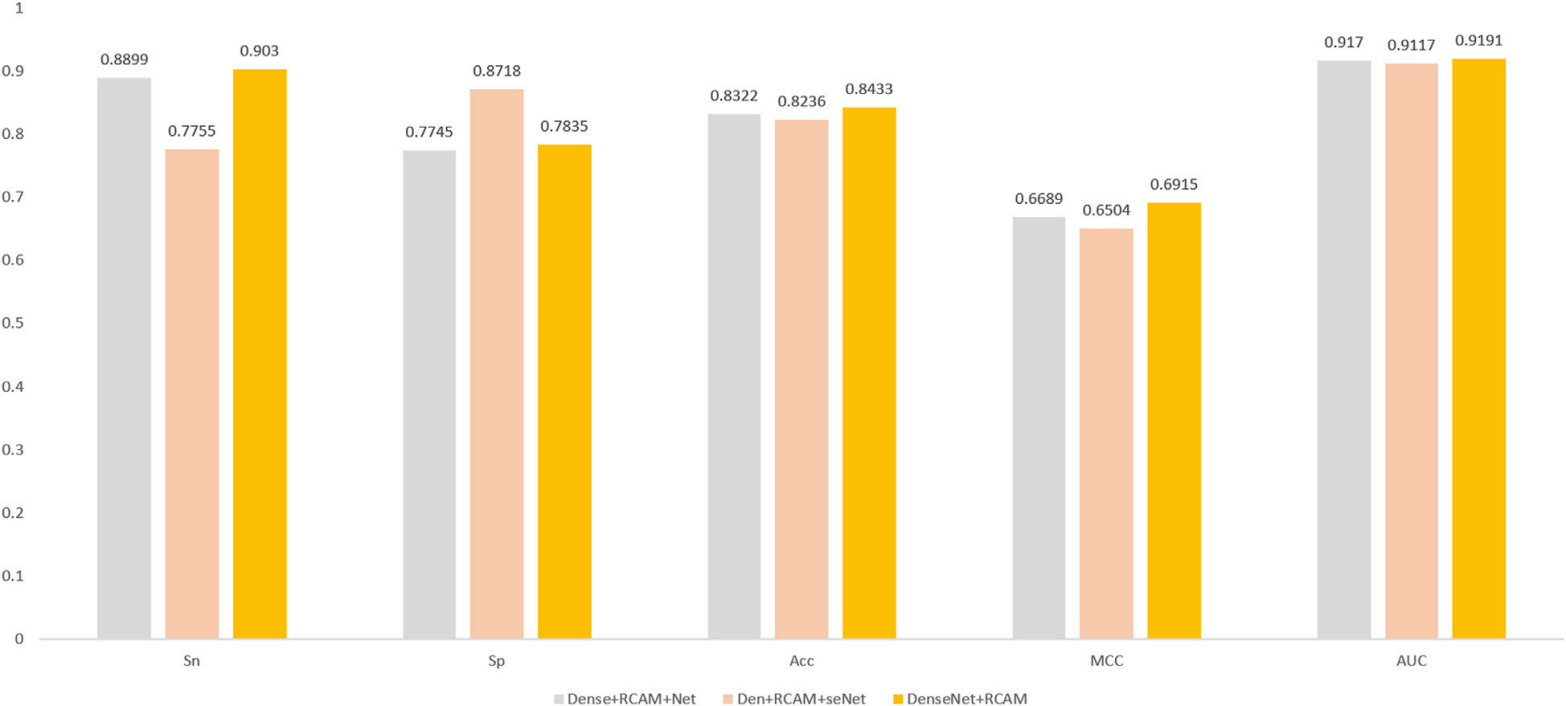
Experimental results for three different network combination structures.

After researching and experimenting with the three combination methods, we arrived at the experimental results shown in Figure 8. Surprisingly, we found that the simplest DenseNet + RCAM combination obtained the best results. We analyzed the effects of these two complex combinations approaches, but found that they led to a complication of the original DenseNet network and triggered an overfitting problem. In contrast, DenseNet + RCAM has less changes in the model structure, which may help to retain DenseNet’s original feature extraction capability and information mobility. This could also explain the better performance of DenseNet + RCAM, because it better balances the complexity and the generalization ability of the model.

### 3.6 Performance of iKcr-DRC on training dataset

**FIGURE 9:**
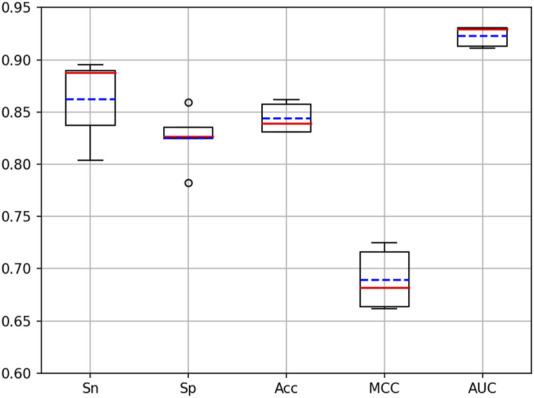
Fluctuations in evaluation metrics for 5-fold cross validation.

**FIGURE 10:**
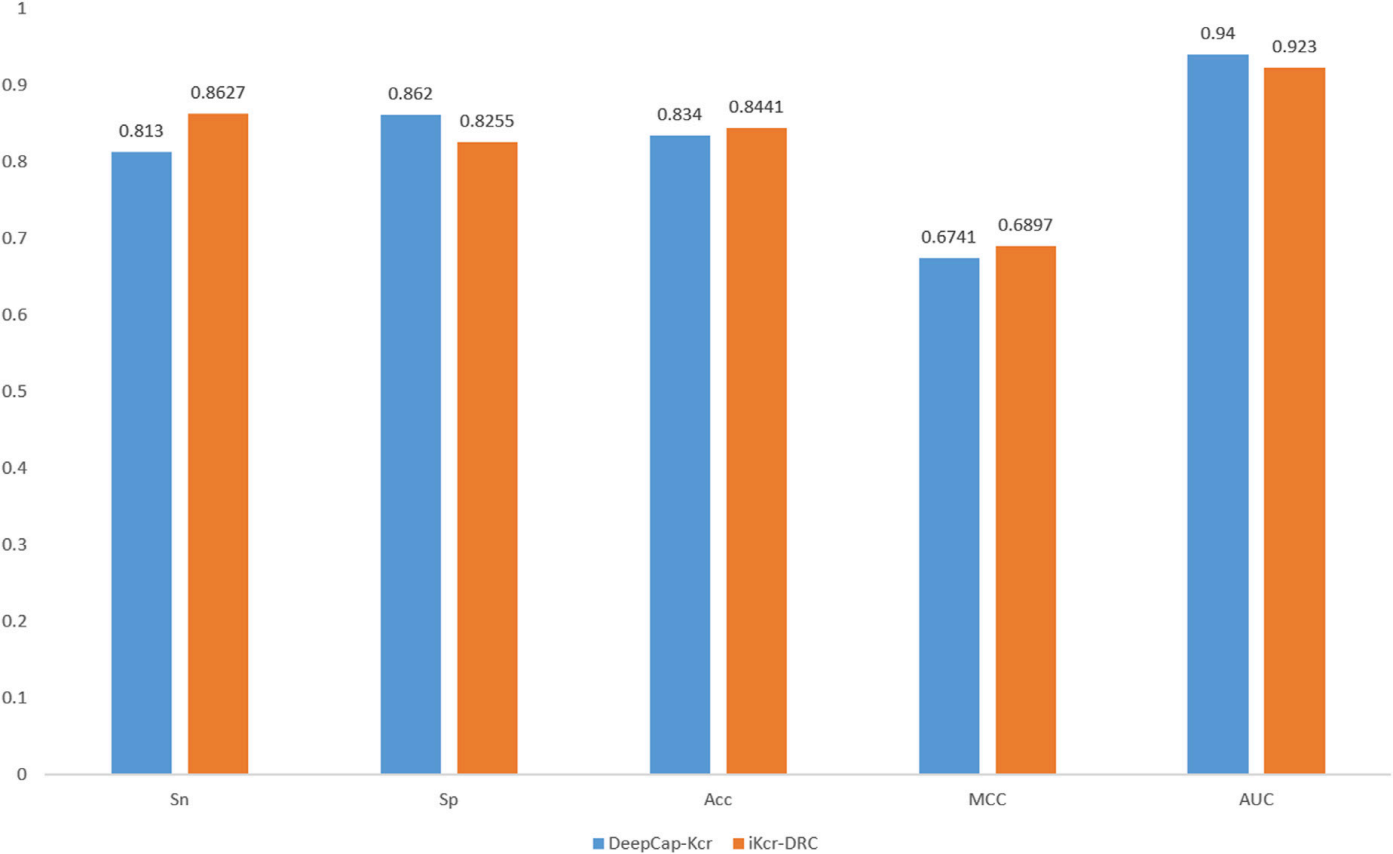
Comparison of Cross-Verification of iKcr-DRC and DeepCap-Kcr.

Cross-validation is a very important means to validate the performance of a model. To validate the performance of the iKcr-DRC model in the task of predicting Kcr site, we performed a 5-fold cross-validation on the training dataset. According to the results shown in Figure 9, the values of other evaluation metrics show relatively stable fluctuations when performing the 5-fold cross-validation, except for two outliers in the Sp metric. This proves that the iKcr-DRC model has more stable and superior performance. In addition, we also compared the average results of the iKcr-DRC model with the DeepCap-Kcr model for 5-fold cross-validation, and the comparison results are displayed in Figure 10. The iKcr-DRC model improved Sn, Acc and MCC by 4.97%, 1.01% and 1.56%, respectively. This indicates that the iKcr-DRC model has good robustness in the task of predicting Kcr site.

### 3.7 Comparison of iKcr-DRC with existing predictors

In recent years, many excellent computational methods have emerged in the field of Kcr sites. To comprehensively evaluate the performance of the iKcr-DRC model, we performed independent tests on benchmark datasets and compared it with existing models. In this way, we can better understand the performance of the iKcr-DRC model on the benchmark dataset. We show the experimental results in Table 4 and found that the iKcr-DRC model significantly outperforms the existing models on four important evaluation metrics (Sn, Acc, MCC and AUC). Specifically, in independent tests, the iKcr-DRC model improved Sn, Acc, MCC, and AUC by 7.9%, 2.03%, 3.55%, and 0.91%, respectively, compared to the most advanced DeepCap-Kcr model. This indicates that the iKcr-DRC model has superior performance and can more accurately and reliably predict Kcr sites. Although the iKcr-DRC model performs very well, it may have a lower Sp metric value compared to other models. This is because we are embedding coding (1-mer), which works better for positive samples, but may slightly reduce the Sp metric value while maintaining high accuracy. Nevertheless, the iKcr-DRC model still has excellent performance and shows higher accuracy in positive sample prediction. And in the field of bioinformatics, accurate prediction of positive samples is a very important task, so our model and coding method are more meaningful in the field of bioinformatics.

**TABLE 4:** Compared with other models on the same independent datasets.

Predictor model	Sn	Sp	Acc	MCC	AUC
Deep-Kcr	0.630	**0.871**	0.751	0.516	0.859
BERT-Kcr	0.801	0.838	0.820	0.640	0.905
DeepCap-Kcr	0.824	0.836	0.823	0.656	0.910
iKcr-DRC	**0.9030**	0.7835	**0.8433**	**0.6915**	**0.9191**

The highest score for each metric is highlighted by bold.

Analyzing other predictors, Deep-Kcr is a predictor developed based on traditional convolutional neural networks, however, traditional convolutional neural networks are relatively low in performance. Although BERT-Kcr utilizes the BERT model and BiLSTM network to capture global features of Kcr site sequences, it is deficient in extracting local feature information. In addition, DeepCap-Kcr employs a capsule network for prediction, which compensates for some of the shortcomings of CNNs, but the performance improvement is limited. In contrast, DenseNet extracts more advanced local information by iteratively utilizing previous features, thus becoming the key to the superior performance of this model.

### 3.8 Comparison of iKcr-DRC with existing predictors in other datasets

Our model employs an adaptive coding method that is capable of accomplishing coding based on the contextual semantic information of the sequence. The core goal of this experiment was to validate the migratory and cross-task adaptation of the iKcr-DRC model, not for predicting other PTM tasks. In this experiment, we did not make any adjustments to the hyperparameters of the iKcr-DRC model, but trained and tested it directly on the training set and independent test set of other PTMs. Despite the differences in the biological mechanisms of different types of PTMs, we hypothesize that certain common structural or functional features may be embedded in the sequence contexts, thus giving the model some potential for cross-task recognition. In addition, to further validate the performance of the iKcr-DRC model, we tested the iKcr-DRC model again on the serine/threonine (S/T) phosphorylation site dataset of Lv et al. (2021b). The benchmark dataset contains 5,387 positive and 5,387 negative samples, and they randomly divide the positive and negative samples into a training set and an independent test set in the ratio of 8:2. The results of the independent tests are shown in Table 5, where our model significantly outperforms the other three prediction models in the Sn, Acc, MCC and AUC metrics. Our model improves the most important MCC metrics by 1.1%–4.3%. It means that the iKcr-DRC model shows excellent robustness in the prediction task of protein post-translational modification and is expected to become the most representative protein post-translational modification prediction model.

**TABLE 5:** Comparison of the serine/threonine (S/T) phosphorylation site dataset with other models.

Predictor model	Sn	Sp	Acc	MCC	AUC
DeepPSP	0.7665	**0.8378**	0.8021	0.606	0. 876
Bert-ST	0.8007	0.7460	0.7984	0.600	0.889
DeepIPs	0.7961	0.8350	0.8063	0.632	0.894
iKcr-DRC	**0.8090**	0.8341	**0.8215**	**0.643**	**0.910**

The highest score for each metric is highlighted by bold.

### 3.9 Prediction of iKcr-DRC in the crotonylation of non-histone lysine

**FIGURE 11:**
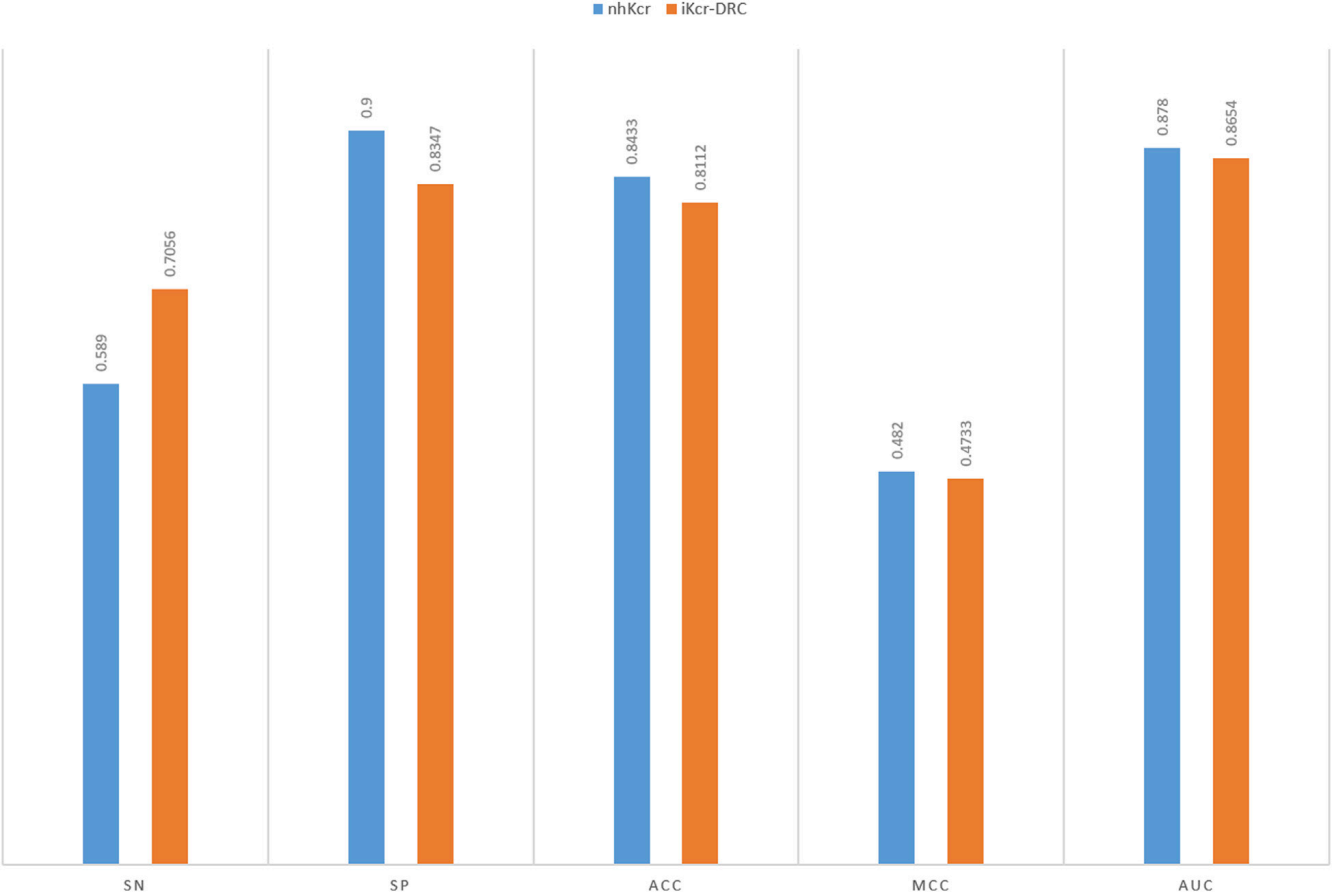
Comparison of the non-histone lysine crotonylation dataset with other models.

Compared to the widely studied lysine crotonylation of histones, non-histone Kcr modifications have shown broader biological significance in the field of functional proteomics. Kcr modifications of non-histone proteins have more diverse regulatory functions in organelles and cellular processes, and its sequence structure is more complex and exhibits a high degree of heterogeneity. As a result, this type of dataset faces significant challenges in the feature learning process, which puts the generalization ability of the predictive model to a great test. Accurate identification of Kcr sites on non-histone proteins not only helps to reveal their potential functions beyond transcriptional regulation, but also sets a higher standard for constructing computational models with good generalization and robustness. Specifically, we tested the iKcr-DRC model on the non-histone lysine crotonylation dataset from Chen Z. et al. (2021). The benchmark dataset contains 15,605 positive samples and 75,111 negative samples, and they randomly divided the positive and negative samples into a training set and an independent test set in the ratio of 8:2. Note that this benchmark dataset is unbalanced. The results of the independent tests are shown in Figure 11, where our model slightly decreases by less than 1% on the most important MCC metric. However, on the Sn metric, our model improves by 11%. In the unbalanced dataset, a higher Sn metric indicates that the model exhibits higher accuracy in the prediction of positive samples. This result re-validates the effectiveness of our model and coding method in positive sample prediction and further demonstrates their importance in the field of bioinformatics. With this extended study, we can more comprehensively assess the performance and potential of the iKcr-DRC model, which will provide an important reference and guidance for further development in the field of non-histone lysine crotonylation research.

## 4 Conclusion

This study is devoted to the development of a new Kcr sites prediction model named iKcr-DRC. we employ the DenseNet network as the core network framework to extract advanced local feature information of Kcr sites. At the same time, we have improved the Channel Attention Mechanism so that it can better highlight important channel feature information. The iKcr-DRC model significantly outperforms other existing models in all evaluation metrics, which proves its excellent expressiveness and performance. We are committed to combining deep learning methods with Kcr site research to drive progress in the Kcr field. The iKcr-DRC model shows potential not only in Kcr site prediction, but also has a value of application in predicting serine/threonine (S/T) phosphorylation sites. This means that the iKcr-DRC model provides researchers with a convenient tool to help advance the field of post-translational modification of proteins.

Although iKcr-DRC performs well on balanced data, it still has shortcomings on unbalanced datasets and requires further research and improvement. Unfortunately, the interpretability of the iKcr-DRC model has not been systematically investigated, which will be one of the core research elements in our follow-up work. As we continue to study the Kcr site, we expect that more advanced deep learning methods will bring more possibilities for Kcr site prediction.

## Data Availability

The data presented in the study are deposited in the https://github.com/weixin7112/iKcr-DRC.
